# Identification of a Prognostic Model Based on Immune-Related Genes of Lung Squamous Cell Carcinoma

**DOI:** 10.3389/fonc.2020.01588

**Published:** 2020-09-02

**Authors:** Rui Li, Xiao Liu, Xi-Jia Zhou, Xiao Chen, Jian-Ping Li, Yun-Hong Yin, Yi-Qing Qu

**Affiliations:** ^1^Department of Pulmonary and Critical Care Medicine, Cheeloo College of Medicine, Qilu Hospital, Shandong University, Jinan, China; ^2^Department of Respiratory Medicine, Tai'an City Central Hospital, Tai'an, China; ^3^Department of Pulmonary and Critical Care Medicine, Qilu Hospital of Shandong University, Jinan, China

**Keywords:** lung squamous cell carcinoma, immune-related genes (IRGs), transcription factors (TFs) mediated IRGs network, a Cox prediction model, prognostic biomarkers

## Abstract

Immune-related genes (IRGs) play considerable roles in tumor immune microenvironment (IME). This research aimed to discover the differentially expressed immune-related genes (DEIRGs) based on the Cox predictive model to predict survival for lung squamous cell carcinoma (LUSC) through bioinformatics analysis. First of all, the differentially expressed genes (DEGs) were acquired based on The Cancer Genome Atlas (TCGA) using the limma R package, the DEIRGs were obtained from the ImmPort database, whereas the differentially expressed transcription factors (DETFs) were acquired from the Cistrome database. Thereafter, a TFs-mediated IRGs network was constructed to identify the candidate mechanisms for those DEIRGs in LUSC at molecular level. Moreover, Gene Ontology (GO), together with Kyoto Encyclopedia of Genes and Genomes (KEGG) pathway enrichment analysis, was conducted for exploring those functional enrichments for DEIRGs. Besides, univariate as well as multivariate Cox regression analysis was conducted for establishing a prediction model for DEIRGs biomarkers. In addition, the relationship between the prognostic model and immunocytes was further explored through immunocyte correlation analysis. In total, 3,599 DEGs, 223 DEIRGs, and 46 DETFs were obtained from LUSC tissues and adjacent non-carcinoma tissues. According to multivariate Cox regression analysis, 10 DEIRGs (including CALCB, GCGR, HTR3A, AMH, VGF, SEMA3B, NRTN, ENG, ACVRL1, and NR4A1) were retrieved to establish a prognostic model for LUSC. Immunocyte infiltration analysis showed that dendritic cells and neutrophils were positively correlated with IRGs, which possibly exerted an important part within the IME of LUSC. Our study identifies a prognostic model based on IRGs, which is then used to predict LUSC prognosis and analyze immunocyte infiltration. This may provide a novel insight for exploring the potential IRGs in the IME of LUSC.

## Introduction

Lung cancer remains a leading factor leading to cancer-related deaths worldwide (1). Lung cancer is associated with a high mortality compared with that of breast cancer (BC), prostate cancer (PCa), colorectal cancer (CRC), and leukemia (1, 2). Lung squamous cell carcinoma (LUSC) occupies about 20–30% non-small cell lung cancer (NSCLC) cases, which causes an annual of 400,000 deaths around the world (3, 4). For TNM stage II LUSC cases, the survival at 5 years is 40%, while that for LUSC cases at pathological TNM stage IV is <5% (5). Moreover, the prognostic biomarkers that can be used in a prediction model for LUSC patients are still lacking (6, 7).

Recently, immunotherapy has been widely recognized to be the efficient therapy for many cancer types (8–11). Recently, Fan et al. had identified reliable markers for predicting the immunotherapy effect on non-small cell lung cancer (NSCLC) (12). Currently, immunotherapy has been considered as the potentially efficient therapy in tumor patients (12). Prat et al. estimated the correlations of immune-related genes (IRGs) expression profiles in squamous NSCLC (sqNSCLC) cases with advanced non-squamous NSCLC (non-sqNSCLC) after PD-1 blockade (13). Furthermore, several clinical studies promote tumor immunology development within LUSC (14). Recently, Li et al. used IRGPs to construct the personalized prognostic model to predict the prognosis for early non-sqNSCLC patients (15). However, the prognostic significance of IRGs and clinical relevance in LUSC have not been illustrated so far.

This study aimed to obtain the differentially expressed genes (DEGs), differentially expressed IRGs (DEIRGs), and differentially expressed TFs (DETFs) in LUSC, so as to establish a Cox prediction model based on the DEIRGs to predict the prognosis for LUSC. The regulatory network between DEIRGs and DETFs possibly exerts an important part in exploring the underlying mechanisms at molecular level. Meanwhile, correlation analysis of immunocytes and risk score also sheds new light on the tumor immune microenvironment (IME) status.

## Materials and Methods

### Clinical Patients and Data Acquisition

Transcriptome RNA-sequencing gene expression profiles were downloaded from TCGA GDC data portal (https://portal.gdc.cancer.gov/), including 502 LUSC as well as 49 non-LUSC tissue specimens. Additionally, FPKM data were downloaded for differential analysis. Meanwhile, IRGs were obtained using the Immunology Database and Analysis Portal (ImmPort) (http://www.immport.org/) (16). Besides, the cancer TF targets were downloaded from Cistrome Project (http://www.cistrome.org/) (17).

### Differential Expression Analysis in LUSC

All transcriptome RNA-Seq data, IRGs, and cancer TF targets were differentially analyzed using the “limma R” package (http://www.bioconductor.org/packages/release/bioc/html/limma.html) (18), according to the thresholds of adjusted false discovery rate (FDR) *P*-value of <0.01 and absolute fold change (log2) of >2. DEIRGs were obtained from DEGs based on the ImmPort database, whereas DETFs were extracted from DEGs using the Cistrome database.

### Functional Analyses for DEIRGs in the Context of LUSC

For exploring the functions among those DEIRGs in terms of their expression profiles, Gene Ontology (GO), together with Kyoto Encyclopedia of Genes and Genomes (KEGG) pathway enrichment analysis, was conducted on DEIRGs using the Database for Annotation, Visualization, and Integrated Discovery (DAVID: https://david.ncifcrf.gov/) (19). Upon GO analysis, a difference of *P* < 0.05 indicated statistical significance. Furthermore, the GOCircle as well as GOChord plotting was obtained using GOplot R package (https://cran.r-project.org/web/packages/GOplot/citation.html) (20). In addition, KEGG pathway enrichment analysis was performed using the “cluster profile R” package (http://www.bioconductor.org/packages/release/bioc/html/clusterProfiler.html) (21), and a difference of P < 0.05 indicated statistical significance. For additionally exploring the associations of DEIRGs with KEGG pathways, the Cytoscape software (version 3.6.1) was employed to construct the pathway-IRGs network for visual analysis.

### Prognosis-Related DEIRGs and TFs Mediated IRGs Regulatory Network

We used the “survival R” package to perform the univariate Cox regression analysis to obtain prognosis-related DEIRGs in LUSC (*P* < 0.05). TFs stand for the significant molecules that can regulate gene expression level directly. Therefore, exploring the mechanism of TFs in the regulation of prognosis-related DEIRGs is of great necessity. The Cistrome Cancer database provides the regulatory interactions between TFs and transcriptomes from TCGA profiles (http://cistrome.org/CistromeCancer/CancerTarget/) (17). To further examine the molecular regulatory mechanisms of prognosis-related DEIRGs, a co-expression network between prognosis-related DEIRGs and DETFs was constructed, according to the thresholds of *p*-value filter of < 0.001 and standard coefficient filter of >0.4.

### Construction of the DEIRGs-Based Prediction Model in LUSC

Univariate Cox regression analysis was carried out for obtaining those prognosis-related DEIRGs as the prognostic biomarkers for multivariate Cox regression analysis (*P* < 0.05). Then, LUSC cases were classified into low or high-risk group according to the median risk score value. The receiver operating characteristics (ROC) curve is based on the specificity and sensitivity of various critical values of continuous diagnostic tests judged according to the binary gold standard (22). To evaluate the specificity and sensitivity of the prognostic model, the ROC curve was performed to examine the signature of DEIRGs (low vs. high risk) on overall survival (OS). Moreover, the area under the ROC curve (AUC) values were determined for evaluating the prognostic model to reveal the prognostic biomarkers in LUSC, with values of 0.5–0.7 representing moderate, 0.7–0.9 representing better, and more than 0.9 representing superior values.

### Correlation Analysis Between Clinical Features and DEIRGs in Prediction Model of LUSC

In order to further explore the correlations between DEIRGs in prediction model and clinical characteristics of LUSC, we used the “beeswarm” R package to analyze the correlations between clinical features (age, gender, pathological T stage, pathological N stage, pathological M stage, and pathological TNM stage) and the expression levels of 10 DEIRGs in the prediction model.

### Infiltration of Immunocytes

Tumor Immune Estimation Resource (TIMER), an online database, is able to analyze and visualize the tumor-infiltrating immunocyte levels (23). It reanalyzes gene expression data of 10,897 TCGA samples from 32 types of cancers for identifying correlation between immunocyte infiltration and other characteristics, incorporating dendritic cells, neutrophils, macrophages, CD4 T cells, CD8 T cells, and B cells (https://zenodo.org/record/57669#.Xeezu9V5uMo).

### Statistical Analysis

The “limma R” package in R software was used for differential analysis, whereas the “GOplot R” and “cluster profile R” packages were adopted for functional enrichment analysis. The prediction model was constructed and applied in univariate as well as multivariate Cox regression analysis. Besides, the “survival ROC R,” “survival R,” “risk Plot R,” and “beeswarm R” packages were adopted to validate the prognostic model in LUSC. The independent *t*-test was performed to validate the heterogeneities in clinical characteristics. A difference of *P* < 0.05 indicated statistical significance.

## Results

### DEGs, DEIRGs, and DETFs

**Figure 1:**
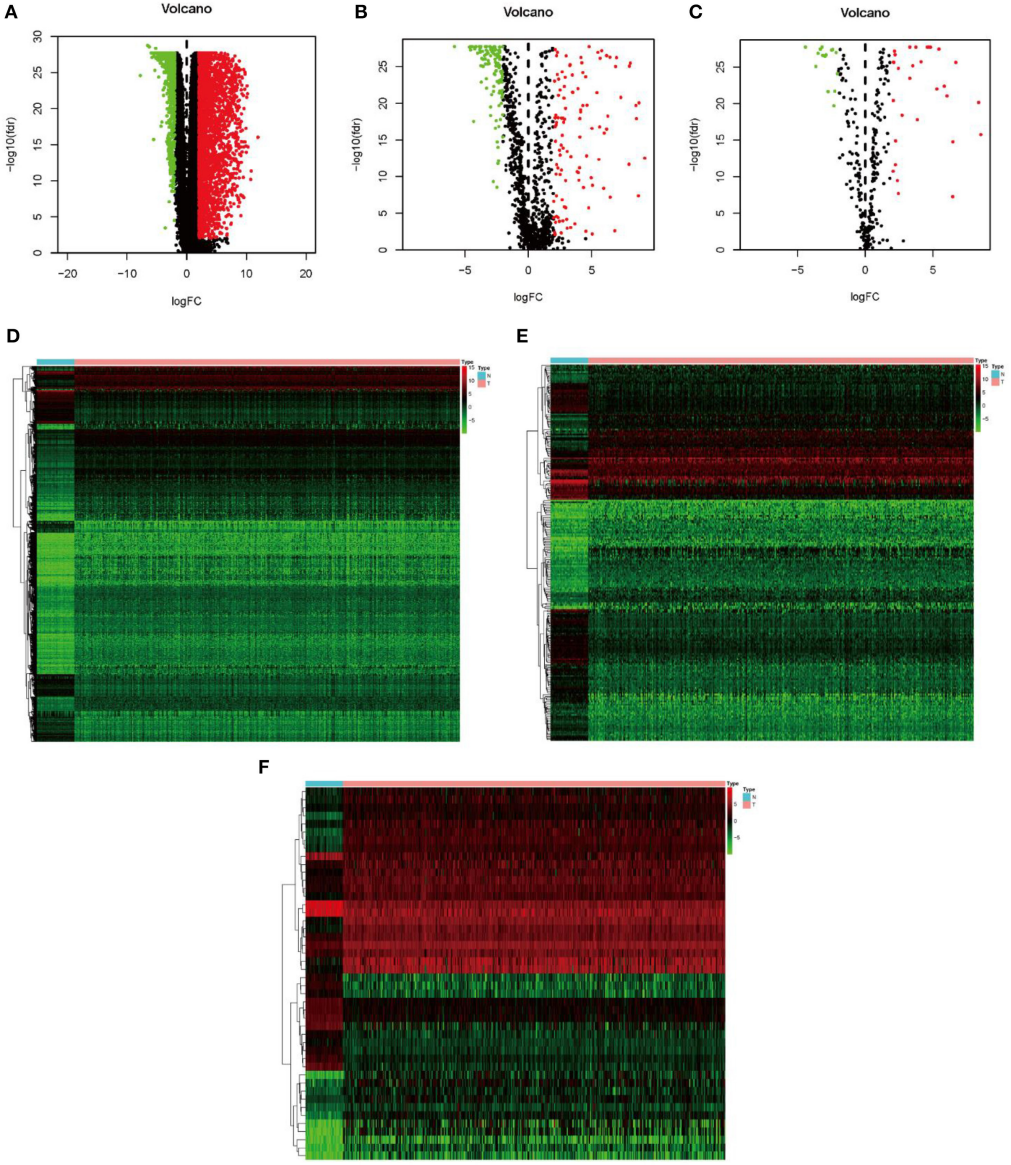
Identification of differential expression genes, IRGs and TFs in LUSC vs. normal tissues. **(A–C)** The volcano plot of differential expression genes, IRGs and TFs in LUSC vs. normal tissues. **(D–F)** The hierarchical clustering heat maps of differential expression genes, IRGs and TFs in LUSC vs. normal tissues.

**Figure 2:**
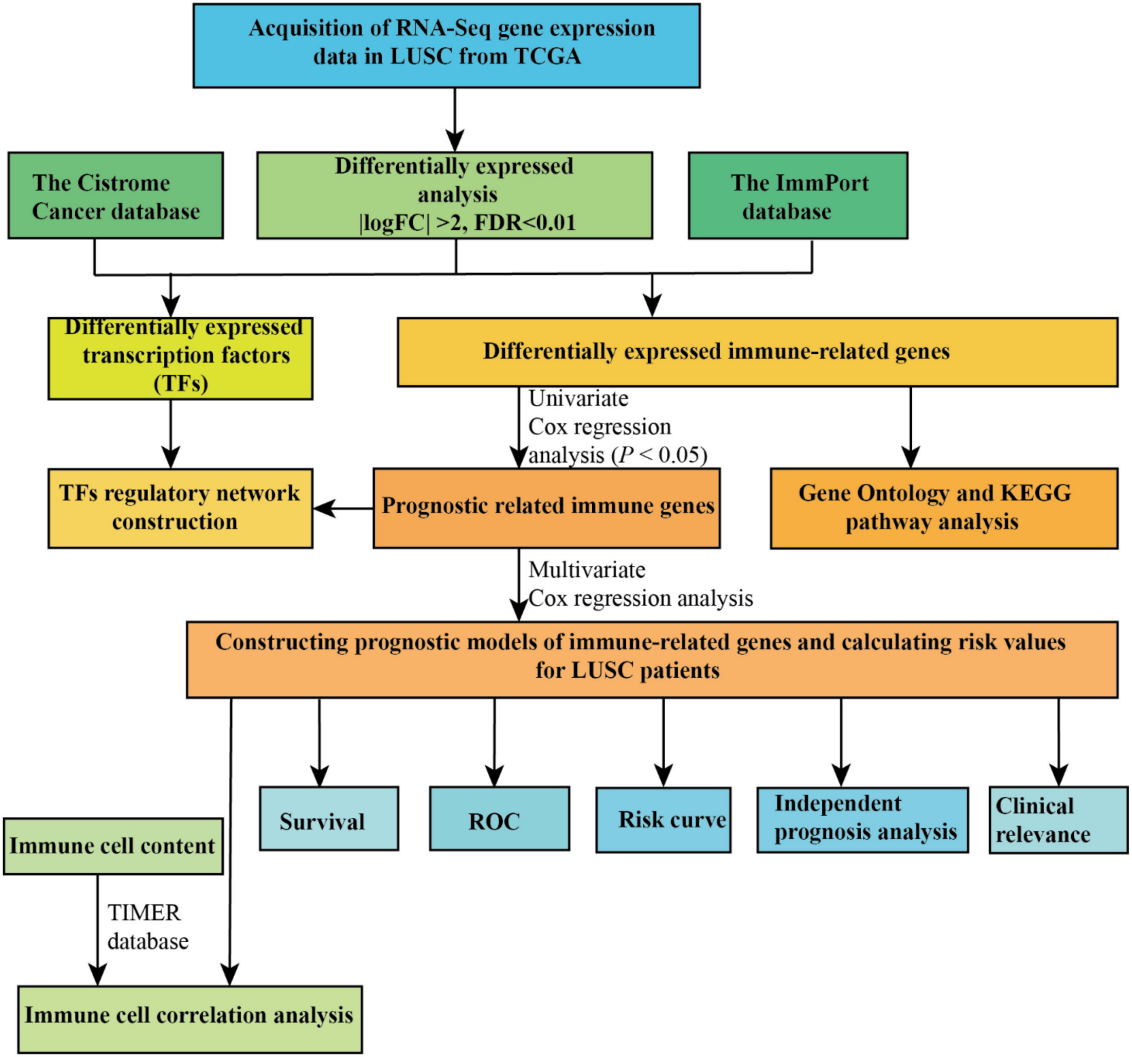
The flow diagram of the whole study.

In the present study, a total of 551 tissues were analyzed, which included 502 LUSC as well as 49 normal tissue specimens. According to the set thresholds (*P* < 0.01 and fold change of >2), 3,599 DEGs, 223 DEIRGs, and 46 DETFs were screened in LUSC as well as non-LUSC tissue specimens. In line with these criteria, we screened 2,598 up-regulated DEGs and 995 down-regulated ones, 110 up-regulated DEIRGs and 113 down-regulated ones, 31 up-regulated DETFs and 15 down-regulated ones. Among them, the top 50 DEGs, DEIRGs, and DETFs are shown in Tables 1–3, respectively. For exhibiting the distributions of all DEGs, DEIRGs, and DETFs at logFC and -log (FDR) dimensions, the volcano plots and heat maps were drawn (Figures 1A–F). Figure 2 presents the flow diagram of this research.

**Table 1:** The top 50 differentially expressed genes in LUSC.

**Gene**	**conMean**	**treatMean**	**logFC**	***p*-value**	**fdr**
AL136369.1	1.98879	0.0600881	−5.04867	1.46E-45	3.01E-41
AC093787.1	2.173824	0.171427	−3.66457	3.72E-39	3.85E-35
LINC02016	2.931133	0.0408282	−6.16575	2.00E-35	1.38E-31
LINC01863	1.503632	0.0785902	−4.25796	2.71E-35	1.40E-31
CHIAP2	6.197897	0.0680397	−6.50926	5.34E-33	1.84E-29
AC008268.1	17.9719	0.2358032	−6.25202	9.24E-33	2.73E-29
AC236972.3	2.186485	0.0599542	−5.18861	1.73E-32	4.48E-29
MND1	0.289446	3.7030027	3.67733	1.70E-30	1.96E-28
RNU5B-4P	1.532508	0.18249	−3.07	2.11E-31	1.96E-28
AL136452.1	2.791136	0.1523629	−4.19527	1.61E-31	1.96E-28
TNXB	11.87023	0.5435242	−4.44886	1.70E-30	1.96E-28
C17orf53	0.360671	4.4624943	3.629094	1.61E-30	1.96E-28
AOC3	73.87922	6.4846345	−3.51007	1.59E-30	1.96E-28
ORC1	0.390146	4.9528996	3.666189	1.85E-30	1.96E-28
ECE2	0.475444	6.3754234	3.745173	1.51E-30	1.96E-28
VEPH1	12.09418	0.6410265	−4.23779	1.59E-30	1.96E-28
NUSAP1	1.93641	23.164864	3.580482	1.85E-30	1.96E-28
TCF21	12.24521	0.5784433	−4.4039	1.31E-30	1.96E-28
NUF2	0.333614	7.8340392	4.553505	1.12E-30	1.96E-28
UBE2T	1.924445	28.589623	3.892977	9.24E-31	1.96E-28
ROBO4	22.11991	1.9320734	−3.51712	1.09E-30	1.96E-28
KIF23	0.509013	6.9497515	3.771186	1.05E-30	1.96E-28
SPAG5	0.786741	9.6333224	3.614073	1.81E-30	1.96E-28
C1orf112	0.505165	2.6353294	2.383156	1.23E-30	1.96E-28
CDT1	0.72442	10.295085	3.828985	1.74E-30	1.96E-28
POLR2H	7.537011	33.727951	2.16188	1.65E-30	1.96E-28
HID1-AS1	1.35454	0.089802	−3.91491	1.40E-30	1.96E-28
RGCC	288.1421	28.362066	−3.34475	1.65E-30	1.96E-28
TGFBR2	115.8615	18.11443	−2.67719	8.19E-31	1.96E-28
HIGD1B	21.27528	1.7726118	−3.58523	1.49E-30	1.96E-28
MCM10	0.186824	3.4332974	4.199846	2.02E-30	1.96E-28
EPAS1	302.4099	33.349552	−3.18077	1.83E-30	1.96E-28
CLIC5	39.51339	1.1414586	−5.11339	9.44E-31	1.96E-28
SELENBP1	111.762	10.323329	−3.43645	1.31E-30	1.96E-28
GGCT	9.148941	40.088046	2.131495	1.49E-30	1.96E-28
PTPRB	14.86132	1.3793306	−3.42952	9.65E-31	1.96E-28
ADH1B	67.03419	2.2531234	−4.8949	1.08E-30	1.96E-28
CCDC34	1.017814	6.1059669	2.584746	1.59E-30	1.96E-28
SFTA1P	82.09988	3.7230534	−4.46282	1.41E-30	1.96E-28
ZWINT	2.229253	22.492483	3.334811	1.32E-30	1.96E-28
CCNF	0.757461	5.0397914	2.73412	1.58E-30	1.96E-28
BIRC5	0.928432	25.691831	4.790369	1.24E-30	1.96E-28
F11	2.491881	0.0953831	−4.70736	7.91E-31	1.96E-28
SLC39A8	89.4114	7.0744202	−3.65977	1.16E-30	1.96E-28
NCAPH	0.575465	10.404005	4.176267	1.20E-30	1.96E-28
ORC6	0.254266	4.3566875	4.098821	1.37E-30	1.96E-28
AQP4	88.754	5.2209159	−4.08744	1.56E-30	1.96E-28
CDH5	49.44781	4.7938158	−3.36666	1.06E-30	1.96E-28
DLC1	30.49691	2.3005572	−3.72861	1.06E-30	1.96E-28
PAICS	5.058223	26.320499	2.379484	2.07E-30	1.96E-28

**Table 2:** Differentially expressed LUSC-specific IRGs.

**ID**	**conMean**	**treatMean**	**logFC**	***p*-value**	**fdr**
HLA-DRB5	461.077	109.62816	−2.0723894	2.71E-22	1.59E-21
ICAM1	200.9799	36.943198	−2.4436708	1.24E-24	1.02E-23
ULBP2	1.50686	9.6289134	2.67582715	6.64E-17	2.26E-16
RAET1L	0.030786	8.0824655	8.03636713	2.54E-27	3.62E-26
PDIA2	0.073611	1.0614969	3.85003002	9.82E-12	2.26E-11
PI3	6.418965	461.90441	6.16910991	8.76E-18	3.23E-17
CAMP	3.875693	0.6317096	−2.6171211	1.37E-25	1.31E-24
PPBP	11.65881	1.0032034	−3.538735	8.03E-27	1.00E-25
CXCL14	5.521033	92.426437	4.06529538	2.82E-19	1.19E-18
CXCL6	1.873474	12.382785	2.72454767	7.67E-05	0.00011
CXCL13	7.549611	63.072959	3.06254743	1.20E-14	3.43E-14
CXCL2	153.9137	11.811675	−3.7038364	1.61E-24	1.29E-23
PF4	3.171328	0.2377575	−3.7375244	5.94E-27	7.68E-26
CXCL3	13.05216	2.2707005	−2.5230792	5.62E-19	2.31E-18
S100A9	353.8853	2142.2339	2.59776246	0.0038027	0.004757
MMP12	0.975359	53.808365	5.78575307	2.25E-29	7.12E-28
SFTPD	614.2878	45.427889	−3.7572644	4.32E-30	2.41E-28
PTGDS	109.6646	20.700635	−2.4053506	3.15E-28	5.75E-27
PGLYRP1	0.900004	0.1497306	−2.5875615	6.34E-18	2.37E-17
S100A7	0.471424	199.23171	8.72320725	1.55E-21	8.27E-21
DEFB126	0.006159	0.4725901	6.26166111	1.24E-19	5.38E-19
PGLYRP3	0.027964	7.0022917	7.96813356	7.21E-27	9.12E-26
S100A2	3.869624	453.25178	6.87197532	7.92E-27	9.90E-26
PGLYRP4	0.067634	2.866368	5.4053312	5.43E-23	3.50E-22
S100A7A	0.00904	3.3194881	8.52035084	2.92E-19	1.23E-18
COLEC12	19.14472	3.0983999	−2.6273512	3.22E-30	2.14E-28
ZC3HAV1L	1.209927	5.077833	2.06929309	1.11E-24	9.22E-24
IL6	52.06098	7.711136	−2.7551872	2.36E-10	4.97E-10
MMP9	10.69609	59.036572	2.46452609	8.00E-15	2.32E-14
A2M	779.4476	80.073835	−3.2830493	1.96E-30	1.96E-28
RBP1	4.821542	27.500384	2.51188523	3.87E-14	1.06E-13
PLAU	12.17388	87.051351	2.83807706	7.23E-24	5.23E-23
PAEP	0.122048	3.3062914	4.75968891	1.11E-06	1.82E-06
SFTPA2	6955.058	386.38136	−4.1699652	3.22E-30	2.14E-28
RBP4	18.01029	3.0610576	−2.5567192	3.99E-26	4.28E-25
SFTPA1	6643.919	304.55793	−4.4472458	1.61E-30	1.96E-28
FABP7	0.024762	6.0551539	7.93389244	8.73E-13	2.17E-12
FABP3	24.57766	5.3883187	−2.1894403	3.86E-19	1.61E-18
FABP4	90.39398	10.227004	−3.1438431	9.78E-28	1.54E-26
CRABP2	5.28148	103.8912	4.29798745	6.32E-26	6.53E-25
CRABP1	0.193769	4.6479257	4.58417545	5.06E-14	1.38E-13
RBP2	3.53304	0.2851016	−3.6313624	6.60E-27	8.42E-26
CTSG	3.970499	0.7948064	−2.3206448	3.81E-18	1.45E-17
PGC	223.8479	9.6219528	−4.5400456	1.63E-29	5.64E-28
TFR2	0.100153	0.9874083	3.30143471	4.30E-27	5.75E-26
CST4	0.00279	0.9936466	8.47641081	3.84E-21	1.95E-20
TLR8	5.311872	1.2317698	−2.1084878	6.26E-25	5.38E-24
WNT5A	3.486577	17.57491	2.33363418	2.90E-12	6.92E-12
MSR1	34.39919	5.0062628	−2.7805688	5.44E-29	1.34E-27
DLL4	10.83279	2.6478642	−2.0325043	2.46E-25	2.26E-24
SLC11A1	15.13747	3.0198257	−2.3255873	1.00E-26	1.22E-25
DMBT1	66.28137	8.9206125	−2.8933888	1.05E-22	6.53E-22
DES	46.13218	2.4958105	−4.2081932	9.47E-30	3.96E-28
MARCO	187.9552	13.295954	−3.8213293	1.50E-29	5.32E-28
TNFSF11	0.131349	0.6009262	2.19378786	2.23E-18	8.65E-18
LTB4R	1.839662	9.386993	2.35122214	2.13E-19	9.07E-19
PTX3	9.998778	1.9020955	−2.3941621	6.55E-13	1.64E-12
MASP1	1.386826	0.2849014	−2.2832521	1.17E-26	1.40E-25
PROC	0.127545	0.8345956	2.71007007	2.36E-19	1.00E-18
NDRG1	38.71135	180.94254	2.2247031	9.88E-19	3.96E-18
RNASE7	0.083332	4.0970218	5.61955547	4.28E-18	1.63E-17
HGF	3.517767	0.8361882	−2.0727603	3.83E-26	4.13E-25
ARRB1	19.75633	2.6517601	−2.8972928	5.41E-30	2.76E-28
PCSK1	0.077937	1.3661507	4.13166332	2.97E-20	1.38E-19
AQP9	14.01407	3.308435	−2.0826552	3.66E-23	2.42E-22
APOH	5.821132	0.4219598	−3.7861222	4.67E-29	1.21E-27
BIRC5	0.928432	25.691831	4.79036933	1.24E-30	1.96E-28
AGER	913.1753	16.384691	−5.8004714	1.06E-30	1.96E-28
CCL14	2.456679	0.2098038	−3.5495966	6.38E-28	1.06E-26
CCL26	0.253326	3.8308785	3.9186095	1.46E-23	1.01E-22
CCL2	113.1869	22.493875	−2.3311034	2.73E-13	7.04E-13
CCL23	9.054335	0.7469719	−3.5994829	3.06E-29	8.82E-28
CCL25	0.040678	0.3132759	2.94512442	1.64E-10	3.49E-10
CCL24	13.27311	2.3991627	−2.4679039	1.43E-09	2.86E-09
FGR	25.27742	5.290182	−2.2564599	4.84E-29	1.24E-27
MIF	14.7758	72.891126	2.30250675	4.59E-29	1.19E-27
OLR1	62.64622	6.9919204	−3.1634667	6.58E-29	1.56E-27
RAC3	1.676118	13.227695	2.9803664	1.75E-28	3.48E-27
CHP2	0.199304	3.057916	3.93950328	1.29E-11	2.95E-11
FOS	333.8072	67.494468	−2.3061738	4.01E-21	2.04E-20
IGHG4	113.2865	646.40241	2.51245651	2.38E-11	5.33E-11
IGKV1-37	0.051562	0.2643369	2.35800204	2.53E-05	3.77E-05
IGKV1D-33	0.42836	1.7998077	2.0709486	0.0033446	0.004203
IGKV2D-28	0.413773	1.8014914	2.12228042	0.0062903	0.00772
IGLV11-55	0.039412	0.2158332	2.45319674	8.86E-05	0.000127
CMA1	0.912088	0.1684654	−2.436721	5.62E-16	1.78E-15
CYR61	219.7772	53.240959	−2.0454331	1.02E-22	6.30E-22
EDN2	1.629824	6.5658989	2.01027659	2.53E-05	3.77E-05
PROK2	1.404653	0.2697099	−2.3807331	3.20E-15	9.54E-15
SEMA3B	15.98977	2.6014242	−2.6197754	6.07E-28	1.02E-26
SEMA3G	12.84195	1.3824059	−3.2156109	3.07E-29	8.82E-28
SEMA4B	10.31778	54.302037	2.39587386	5.90E-25	5.09E-24
SLIT2	13.30398	2.3227635	−2.5179442	1.36E-28	2.82E-27
TNC	16.76456	67.140469	2.00176783	1.41E-07	2.46E-07
C5AR1	37.98875	8.0161238	−2.2445957	2.45E-28	4.65E-27
CCRL2	7.041071	1.445888	−2.2838392	1.67E-29	5.73E-28
CX3CR1	5.167159	0.5712854	−3.1770877	3.40E-27	4.67E-26
EDNRB	32.29231	1.87733	−4.1044363	1.43E-30	1.96E-28
FPR1	24.59865	5.0476023	−2.2849091	6.14E-23	3.92E-22
FPR2	7.519719	0.7269767	−3.3706979	3.36E-27	4.63E-26
GPR17	1.114164	0.1314182	−3.0837243	2.85E-27	4.01E-26
LTB4R2	0.460114	1.9873162	2.1107577	2.18E-19	9.29E-19
PLXNB3	0.339127	3.2805048	3.27401847	5.72E-27	7.42E-26
ADM2	0.463038	3.4662184	2.90416101	3.15E-25	2.84E-24
AGRP	4.924205	0.2325856	−4.4040572	2.07E-30	1.96E-28
AMH	0.13159	0.942215	2.84000501	8.13E-07	1.35E-06
APLN	23.412	3.00256	−2.9629829	1.03E-19	4.53E-19
ARTN	0.188148	8.4091957	5.48202581	4.84E-29	1.24E-27
BMP2	22.54047	4.9700208	−2.181194	7.99E-25	6.77E-24
BMP5	7.604228	1.4532498	−2.3875191	2.60E-27	3.70E-26
BMP7	0.863562	17.007779	4.29975063	3.90E-21	1.99E-20
CALCB	0.018958	0.2403375	3.66421158	0.000197	0.000274
CGA	0.004451	0.5006056	6.81336953	0.0019182	0.002461
CGB7	0.043046	0.3288359	2.9334072	1.00E-20	4.88E-20
CHGA	0.135288	4.4348736	5.03478987	0.0053207	0.006571
CHGB	0.085331	2.6634662	4.96408972	6.77E-11	1.47E-10
CMTM2	1.853074	0.4255054	−2.1226714	1.88E-21	9.90E-21
CSF3	39.48564	2.0020425	−4.3017836	7.05E-19	2.87E-18
DKK1	1.896679	8.8747812	2.226236	1.78E-05	2.68E-05
FGF11	0.033473	0.6242029	4.2209609	2.21E-27	3.21E-26
FGF12	0.347641	1.6173135	2.2179291	2.32E-08	4.25E-08
FGF18	1.920716	0.4252406	−2.175293	4.36E-26	4.66E-25
FGF19	0.006679	3.8373226	9.16618982	1.07E-13	2.84E-13
FGF8	0.036014	0.2943497	3.03091193	4.61E-05	6.73E-05
GAL	0.05919	5.1206915	6.43485126	3.00E-28	5.54E-27
GAST	0.020864	3.2728535	7.29341768	2.74E-22	1.61E-21
GDF10	11.18805	0.4802897	−4.5419097	2.59E-30	2.02E-28
GDNF	0.034377	0.8466851	4.62231808	3.53E-21	1.81E-20
GPI	19.82174	86.319584	2.12260465	1.33E-29	4.96E-28
GREM1	0.452455	5.1376902	3.50527377	5.04E-23	3.26E-22
GREM2	0.881029	0.213174	−2.0471577	2.66E-23	1.79E-22
IFNE	0.023322	0.3698422	3.98716901	1.49E-10	3.18E-10
IL11	0.2357	1.4544656	2.62546653	2.38E-19	1.01E-18
IL19	0.009421	0.3151672	5.0641215	7.94E-10	1.61E-09
IL23A	0.705467	4.0015916	2.50392301	2.24E-23	1.52E-22
INHA	0.092499	1.6216373	4.13187128	1.70E-20	8.09E-20
INHBE	0.080694	0.5566857	2.78633466	9.09E-19	3.66E-18
JAG1	8.342649	43.423872	2.3799109	1.34E-22	8.19E-22
KL	3.189386	0.320923	−3.3129795	1.72E-29	5.86E-28
LEFTY2	1.2978	0.131131	−3.3069875	1.02E-28	2.23E-27
MDK	17.16142	108.6156	2.66199044	5.12E-26	5.37E-25
NDP	0.083266	0.3699254	2.15144096	0.0001198	0.000169
NPPC	0.102034	6.4303417	5.97777863	3.10E-17	1.09E-16
NRTN	0.42792	1.8945531	2.14644486	4.41E-16	1.40E-15
NTS	2.922934	256.00821	6.45262894	3.66E-08	6.63E-08
OGN	11.89253	0.7461729	−3.9944021	6.65E-29	1.57E-27
POMC	0.645712	5.0113501	2.95623682	1.04E-06	1.71E-06
PTHLH	0.474844	58.687804	6.94946355	3.39E-28	6.12E-27
REG1A	0.011046	0.2256665	4.35254251	1.60E-09	3.18E-09
RETN	26.71932	1.0816423	−4.6265878	3.78E-30	2.23E-28
SCG2	0.673358	6.8328843	3.34304839	0.0006537	0.000871
SCGB3A1	340.4085	35.154133	−3.2755009	1.25E-23	8.69E-23
SLURP1	0.027479	1.2987794	5.56270309	1.70E-13	4.45E-13
SPP1	19.69514	452.25766	4.52123351	2.80E-25	2.55E-24
TG	0.064614	0.3917236	2.59991949	1.57E-15	4.78E-15
TNFSF13	19.26061	4.7280304	−2.0263422	2.96E-30	2.11E-28
UCN2	0.034901	2.5094825	6.16798312	1.66E-28	3.34E-27
VGF	0.044767	0.7533296	4.07276254	1.13E-16	3.78E-16
ACVR1C	0.114121	0.7988812	2.80741073	3.32E-22	1.92E-21
ACVRL1	38.01144	4.6870551	−3.0196801	8.01E-31	1.96E-28
ADRB1	9.178733	0.8539406	−3.4260873	1.51E-28	3.08E-27
ADRB2	11.55157	1.5885658	−2.8622923	1.05E-29	4.25E-28
AGTR1	2.398264	0.3281341	−2.8696331	3.23E-29	9.14E-28
AGTR2	10.89749	1.1084088	−3.2974335	5.33E-25	4.65E-24
ANGPT1	8.056934	1.2371929	−2.7031604	8.30E-29	1.89E-27
ANGPTL1	4.07565	0.3954179	−3.3655799	2.83E-30	2.07E-28
AVPR2	1.210134	0.2127857	−2.5076941	3.26E-26	3.58E-25
CALCRL	31.44559	5.2338716	−2.5869074	1.72E-27	2.56E-26
CSF3R	15.61837	3.2362367	−2.270855	5.17E-27	6.79E-26
ENG	111.7984	23.420986	−2.2550262	3.75E-30	2.23E-28
FGFR3	7.514727	34.279517	2.18955411	2.10E-11	4.73E-11
FGFR4	16.22647	2.093487	−2.9543695	1.33E-29	4.96E-28
FLT4	6.553111	1.3946479	−2.232279	6.26E-28	1.05E-26
GALR2	0.056636	0.3470297	2.61525621	5.63E-12	1.32E-11
GCGR	0.012172	0.3952067	5.02096267	6.83E-18	2.54E-17
HNF4G	0.144068	0.6783255	2.23522365	9.36E-13	2.32E-12
HTR3A	0.058628	0.9644891	4.04009594	2.60E-12	6.23E-12
HTR3C	1.12333	0.1739655	−2.6909089	1.00E-25	9.85E-25
IL12RB2	0.157606	1.0603025	2.75008257	4.12E-15	1.22E-14
IL1RL1	12.31321	0.9700207	−3.6660481	4.54E-28	7.88E-27
IL1RL2	0.356559	1.5434502	2.11394595	4.01E-18	1.53E-17
IL20RB	0.334829	16.959207	5.66250039	4.22E-28	7.41E-27
IL22RA2	0.059908	0.4350077	2.86021389	3.31E-19	1.39E-18
IL31RA	0.048264	0.496303	3.36218938	6.63E-14	1.79E-13
IL3RA	22.1212	4.6459548	−2.2513828	2.53E-29	7.63E-28
IL5RA	1.221382	0.1871493	−2.7062533	7.09E-24	5.13E-23
KDR	18.30759	3.376128	−2.4390006	9.03E-29	2.01E-27
LEPR	6.218121	1.0129005	−2.6179862	1.96E-29	6.46E-28
LGR4	1.755462	8.5709768	2.28760862	1.27E-25	1.22E-24
LIFR	14.38279	3.3477143	−2.1030953	8.69E-26	8.69E-25
NGFR	1.334232	8.4440305	2.6619221	8.37E-08	1.48E-07
NPR1	17.17307	1.2954758	−3.7285939	1.01E-30	1.96E-28
NR0B1	0.013368	5.448542	8.67094443	2.29E-08	4.21E-08
NR0B2	4.127285	0.8196907	−2.3320415	2.36E-28	4.49E-27
NR3C2	4.652115	0.9041322	−2.3632811	1.07E-28	2.31E-27
NR4A1	64.22084	9.6411929	−2.735758	3.56E-23	2.35E-22
NR4A2	17.96171	4.4469599	−2.0140337	1.07E-16	3.58E-16
NR4A3	13.16555	1.6840739	−2.9667404	6.44E-21	3.19E-20
NR5A1	0.009207	0.5656858	5.94115282	2.06E-09	4.06E-09
NR5A2	1.380175	0.2935232	−2.2333049	3.09E-23	2.06E-22
OPRK1	0.059067	0.4938854	3.06376221	0.0007962	0.001053
PTH1R	3.487933	0.5849558	−2.5759728	1.24E-29	4.78E-28
PTH2R	0.04934	1.1430188	4.53395516	0.0021887	0.002795
RORC	5.43979	1.1361492	−2.2593987	9.43E-25	7.89E-24
RXFP1	1.799126	0.3225014	−2.4799191	3.36E-27	4.63E-26
RXRG	2.374553	0.3050428	−2.9605726	1.75E-28	3.48E-27
S1PR1	53.92726	5.198261	−3.3749137	1.21E-30	1.96E-28
SCTR	6.672413	1.1492864	−2.5374702	6.53E-27	8.35E-26
SSTR1	4.071218	0.3735964	−3.4459081	1.62E-28	3.27E-27
TEK	20.30388	1.4018787	−3.8563218	9.44E-31	1.96E-28
TGFBR2	115.8615	18.11443	−2.6771903	8.19E-31	1.96E-28
TIE1	16.35046	2.5457047	−2.6831939	3.44E-30	2.19E-28
TNFRSF10C	3.589903	0.8587616	−2.0636151	1.69E-24	1.35E-23
TNFRSF18	1.303109	14.476521	3.47368513	1.50E-25	1.43E-24
TNFRSF25	1.140956	5.1233473	2.16684331	8.68E-20	3.84E-19
TUBB3	0.10875	1.2231949	3.49156862	4.42E-23	2.88E-22
VIPR1	16.39261	1.5679781	−3.3860679	5.53E-28	9.39E-27
ICAM2	10.66629	2.1235578	−2.328503	2.05E-29	6.67E-28
SHC3	2.660188	0.4328611	−2.6195523	1.18E-28	2.51E-27
SH2D1B	0.946486	0.2338434	−2.0170378	7.89E-20	3.51E-19
CBLC	1.349684	24.094321	4.1579992	3.25E-28	5.90E-27
PDK1	0.900902	4.2255279	2.22968943	1.68E-28	3.37E-27
TRGJP2	6.410727	1.2902415	−2.3128469	2.20E-24	1.73E-23

**Table 3:** Differentially expressed TFs.

**ID**	**conMean**	**treatMean**	**logFC**	***p*-value**	**fdr**
BCL11A	0.564995	5.573467	3.302266	3.95E-25	3.51E-24
BRCA1	0.715878	3.122877	2.125091	1.59E-27	2.38E-26
CBX2	0.657288	7.539504	3.519873	5.22E-27	6.84E-26
CDX2	0.003056	0.270279	6.466585	2.99E-08	5.46E-08
CENPA	0.277394	7.535176	4.76363	1.03E-30	1.96E-28
E2F1	2.77481	11.86825	2.096646	1.48E-27	2.23E-26
E2F7	0.123771	3.109288	4.650835	2.28E-30	1.97E-28
EMX1	0.005446	0.358422	6.040421	1.47E-22	8.91E-22
EPAS1	302.4099	33.34955	-3.18077	1.83E-30	1.96E-28
ERG	9.45181	1.810232	-2.38442	9.89E-30	4.06E-28
EZH2	0.904531	8.895519	3.297837	1.94E-30	1.96E-28
FLI1	8.69851	1.975634	-2.13845	4.00E-29	1.08E-27
FOS	333.8072	67.49447	-2.30617	4.01E-21	2.04E-20
FOXA2	14.60491	1.667552	-3.13065	1.25E-28	2.64E-27
FOXM1	0.857509	20.68245	4.592112	1.74E-30	1.96E-28
GATA6	11.25897	2.044409	-2.46132	1.31E-29	4.93E-28
H2AFX	10.15427	45.57329	2.166102	2.11E-29	6.81E-28
HNF1B	4.134758	0.37293	-3.47083	5.55E-29	1.36E-27
HNF4G	0.144068	0.678326	2.235224	9.36E-13	2.32E-12
HOXA9	0.107939	0.590502	2.451718	1.07E-08	2.01E-08
HOXB13	0.006008	2.234528	8.538856	5.14E-17	1.77E-16
HOXB7	1.658101	10.96895	2.725822	9.06E-20	4.00E-19
HOXC11	0.003249	1.075878	8.37151	1.30E-21	6.97E-21
HOXC9	0.120816	1.739933	3.848151	3.95E-19	1.64E-18
LHX2	0.012917	1.339701	6.696443	1.62E-27	2.42E-26
LMNB1	4.194354	19.46207	2.214144	9.73E-29	2.14E-27
MYBL2	1.383085	39.37181	4.831201	2.00E-30	1.96E-28
NCAPG	0.389631	5.24307	3.750232	2.41E-30	1.98E-28
NFE2	2.345071	0.566439	-2.04964	7.79E-26	7.88E-25
NR4A1	64.22084	9.641193	-2.73576	3.56E-23	2.35E-22
NR5A2	1.380175	0.293523	-2.2333	3.09E-23	2.06E-22
PAX3	0.002614	0.233341	6.480211	5.45E-16	1.72E-15
RBP2	3.53304	0.285102	-3.63136	6.60E-27	8.42E-26
RXRG	2.374553	0.305043	-2.96057	1.75E-28	3.48E-27
SALL4	0.058452	1.022483	4.128681	1.26E-27	1.94E-26
SCML2	0.228445	1.192228	2.38374	1.58E-10	3.35E-10
SNAI2	5.61899	23.77576	2.081109	7.12E-22	3.93E-21
SOX17	6.052244	0.613681	-3.30191	8.42E-30	3.66E-28
SOX2	2.181036	85.96764	5.300708	1.48E-23	1.02E-22
SOX9	3.284094	13.75398	2.066282	7.94E-12	1.84E-11
TAL1	3.049298	0.413054	-2.88408	1.05E-28	2.27E-27
TCF21	12.24521	0.578443	-4.4039	1.31E-30	1.96E-28
TFAP2A	0.158501	6.923488	5.448932	8.24E-30	3.61E-28
TFAP2C	2.540361	13.91772	2.453817	1.32E-26	1.56E-25
TP63	1.27116	73.18094	5.847251	5.80E-24	4.26E-23
TP73	0.806192	3.82784	2.247336	3.90E-16	1.25E-15

### Functional Analyses for DEIRGs

**Figure 3:**
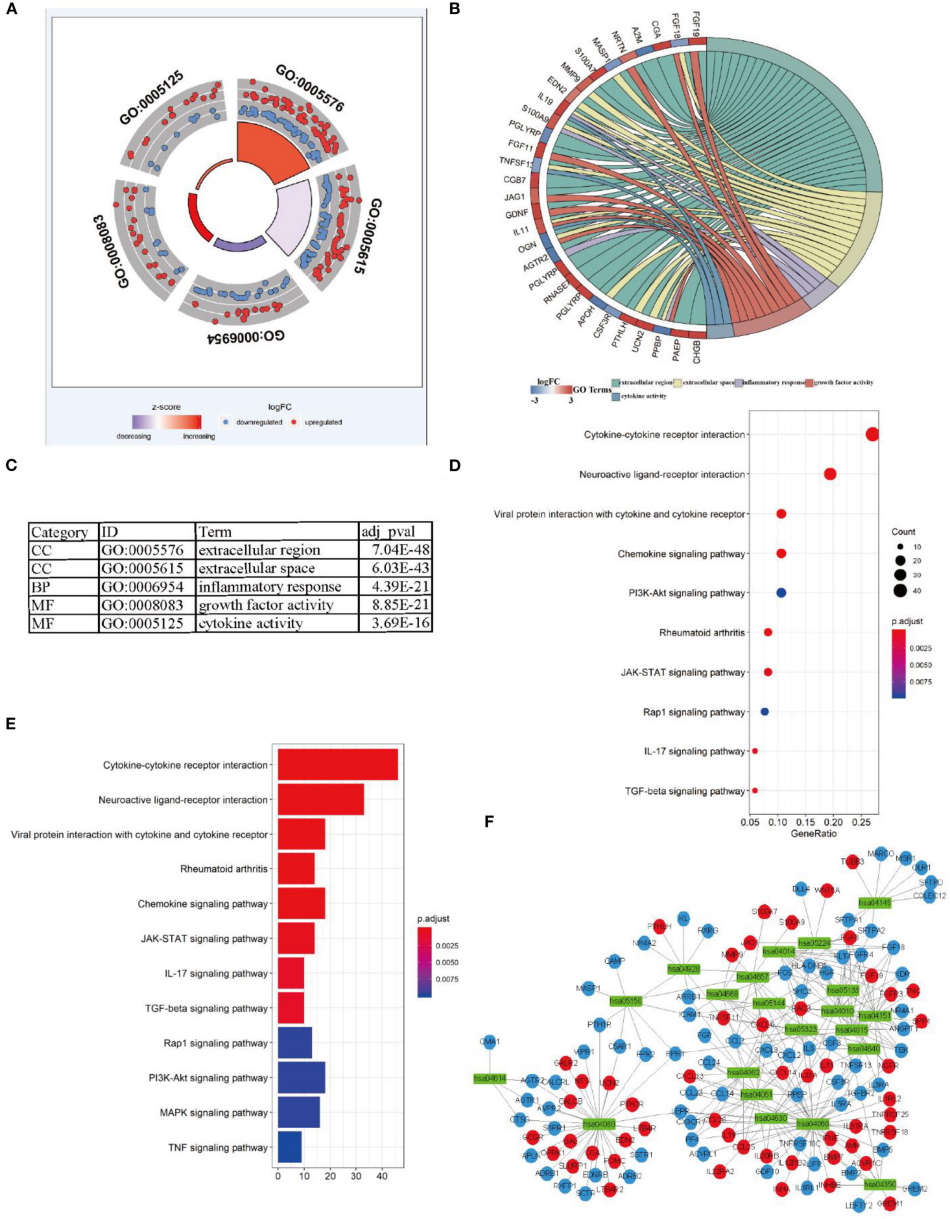
Functional enrichment analysis of differential expression IRGs in LUSC. **(A)** The outer circle shows the expression (logFC) of differential expression IRGs in each enriched GO terms: red dots which were on each Go terms indicated the up-regulation differential expression IRGs. Blue dots indicated the down-regulation differential expression IRGs. The inner-circle shows the prominence of GO terms (log10-adjusted *P*-values). **(B)** The circle represents the relationship between statistically top 30 differential expression IRGs and their GO terms. **(C)** The top five most significant GO terms and their annotations. **(D)** The top 10 pathways which were enriched in differential expression IRGs were showed in the dot plot. **(E)** The top 12 pathways which were enriched in differential expression IRGs were showed in the barplot. **(F)** the significantly statistically different 21 pathways were used Cytoscape software for constructing a pathway-IRG network with differential expression IRGs. The green rectangles indicate the pathways, the red circles indicate the up-regulation differential expression IRGs, the blue circles indicate the down-regulation differential expression IRGs.

For explaining biological functions of DEIRGs in LUSC patients, functional enrichment analyses were conducted. According to GO analysis results, 5 GOs displayed significant difference (*P* < 0.05), among which, “GO: 0005576 extracellular region” was the most significant GO term (Figures 3A,C). Figure 3B shows the correlations between the top 30 statistically significant DEIRGs and corresponding GO terms. Furthermore, the “cluster profile R” package was used for KEGG pathway enrichment analyses. The dot plot shows the 10 most significant pathways with the highest enrichment levels of DERGs within the KEGG database (Figure 3D). In addition, the bar plot indicates the 12 most significant pathways with the highest enrichment levels of DEIRGs within the KEGG database (Figure 3E). Those 21 statistically significant pathways in the KEGG database were selected to construct the “pathway-DEIRGs” network (Figure 3F). The 21 statistically significant pathways in the KEGG database are shown in Table 4. A difference of *P* < 0.05 indicated statistical significance.

**Table 4:** KEGG Pathway analysis of differential expression IRGs in LUSC.

**ID**	**Description**	**Count**	***p*-value**	***p*.adjust**	***q*-value**
hsa04060	Cytokine-cytokine receptor interaction	46	8.05E-28	1.48E-25	1.21E-25
hsa04080	Neuroactive ligand-receptor interaction	33	1.38E-13	1.27E-11	1.04E-11
hsa04061	Viral protein interaction with cytokine and cytokine receptor	18	2.57E-12	1.58E-10	1.29E-10
hsa05323	Rheumatoid arthritis	14	8.87E-09	4.08E-07	3.34E-07
hsa04062	Chemokine signaling pathway	18	1.03E-07	3.80E-06	3.11E-06
hsa04630	JAK-STAT signaling pathway	14	9.11E-06	0.0002793	0.0002285
hsa04657	IL-17 signaling pathway	10	2.79E-05	0.000705	0.0005768
hsa04350	TGF-beta signaling pathway	10	3.07E-05	0.000705	0.0005768
hsa04015	Rap1 signaling pathway	13	0.00056668	0.0097149	0.0079475
hsa04151	PI3K-Akt signaling pathway	18	0.00056926	0.0097149	0.0079475
hsa04010	MAPK signaling pathway	16	0.00058078	0.0097149	0.0079475
hsa04668	TNF signaling pathway	9	0.00065132	0.0099868	0.00817
hsa05224	Breast cancer	10	0.00121214	0.0171564	0.0140353
hsa04614	Renin-angiotensin system	4	0.00132305	0.0173887	0.0142253
hsa04014	Ras signaling pathway	13	0.00142622	0.017495	0.0143123
hsa04928	Parathyroid hormone synthesis, secretion and action	8	0.00194247	0.0223384	0.0182746
hsa05144	Malaria	5	0.00383322	0.041489	0.0339412
hsa05150	Staphylococcus aureus infection	7	0.0044752	0.0457465	0.0374242
hsa04640	Hematopoietic cell lineage	7	0.00473805	0.0458843	0.0375369
hsa04145	Phagosome	9	0.0053595	0.0493074	0.0403373
hsa05133	Pertussis	6	0.0056862	0.0498219	0.0407582

### Univariate Cox Regression Analysis and Regulatory Network of Prognosis-Related DEIRGs and DETFs

**Figure 4:**
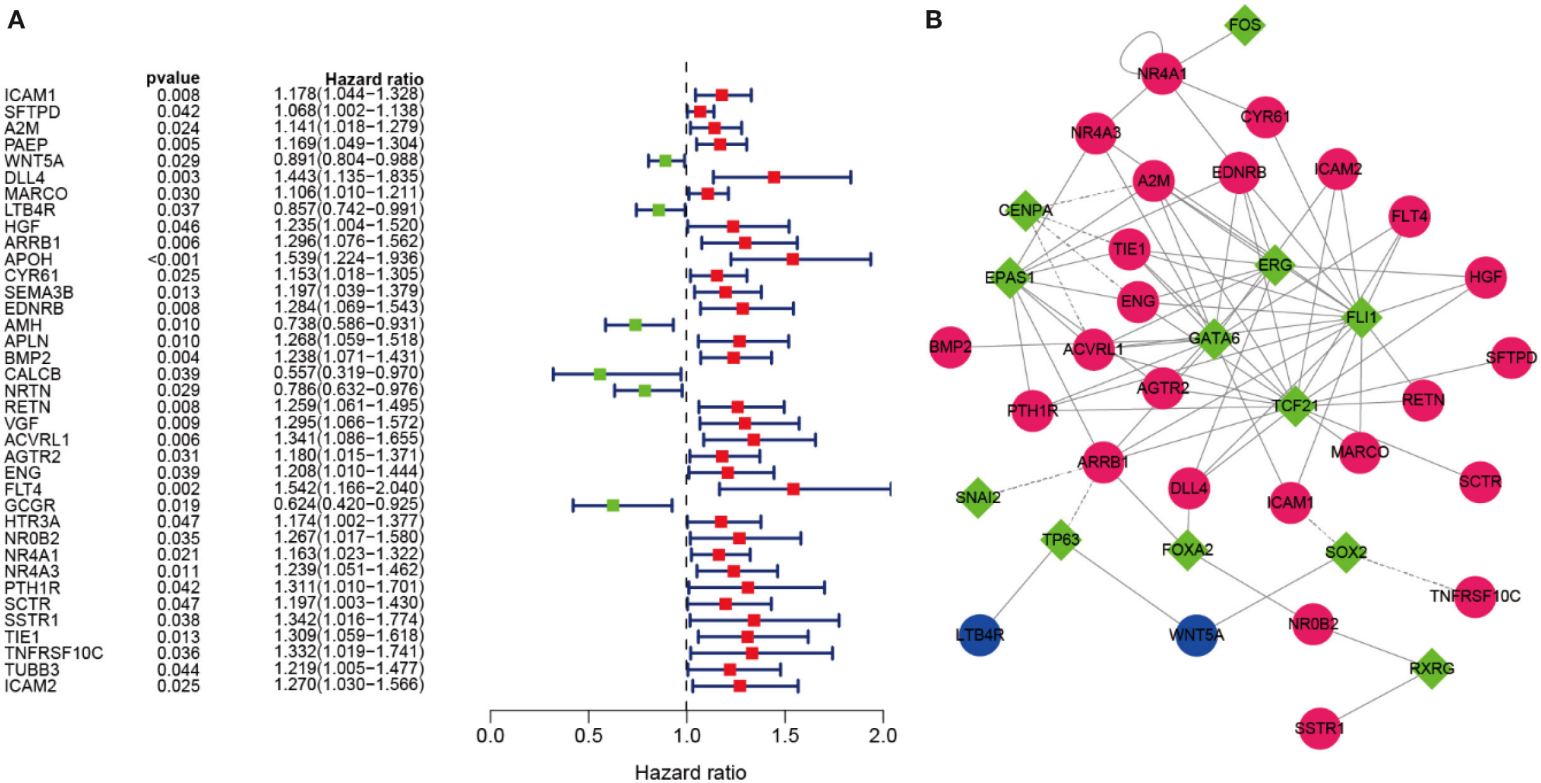
OS-related DEIRGs and TFs-IRGs regulatory network. **(A)** The forest map of OS-related DEIRGs in LUSC. Red and green dots indicate the high and low-risk, respectively. **(B)** Regulatory network between prognosis-related DEIRGs and DETFs in LUSC. The red and blue circles indicate the high and low-risk DEIRGs, respectively. The green diamonds indicate DETFs. Solid and dashed lines in the network showed that there is a positive and negative correlation between prognosis-related DEIRGs and DETFs.

Univariate Cox regression analysis was analyzed to identify prognosis-related DEIRGs in LUSC (*P* < 0.05). Then, 37 OS-related DEIRGs were identified, incorporating 31 high-risk DEIRGs and 6 low-risk DEIRGs (Figure 4A). For exploring the molecular mechanism of prognosis-related DEIRGs, we constructed the TFs-IRGs regulatory network. A total of 26 prognosis-related DEIRGs and 13 DETFs were shown in the network (Figure 4B). As shown in Figure 4B, CENPA had a negatively relationship with A2M, TIE1, ENG, and ACVRL1. ARRB1 had a negatively relationship with TP63, SNAI2. SOX2 had a negatively relationship with ICAM1 and TNFRSF10C. The coefficient filter >0.4 and the *p*-value filter < 0.001 were set as the threshold to indicate statistical significance. Table 5 shows the regulatory network between DETFs and prognosis-related DEIRGs in LUSC.

**Table 5:** Correlation analysis between TFs and IRGs in LUSC.

**TF**	**ImmuneGene**	**cor**	***p*-value**	**Regulation**
CENPA	A2M	−0.4136	3.07E-19	Negative
CENPA	ACVRL1	−0.4029	3.01E-18	Negative
CENPA	ENG	−0.4025	3.27E-18	Negative
CENPA	TIE1	−0.4228	4.06E-20	Negative
EPAS1	A2M	0.42186	5.01E-20	Positive
EPAS1	ARRB1	0.40984	6.89E-19	Positive
EPAS1	EDNRB	0.49683	3.01E-28	Positive
EPAS1	ACVRL1	0.42222	4.62E-20	Positive
EPAS1	AGTR2	0.40382	2.46E-18	Positive
EPAS1	ENG	0.44962	7.77E-23	Positive
EPAS1	NR4A3	0.4066	1.37E-18	Positive
EPAS1	PTH1R	0.49468	5.55E-28	Positive
EPAS1	TIE1	0.48626	5.81E-27	Positive
ERG	A2M	0.48924	2.55E-27	Positive
ERG	DLL4	0.40045	4.96E-18	Positive
ERG	HGF	0.41438	2.59E-19	Positive
ERG	EDNRB	0.46326	2.58E-24	Positive
ERG	ACVRL1	0.46495	1.68E-24	Positive
ERG	AGTR2	0.45101	5.53E-23	Positive
ERG	ENG	0.4115	4.83E-19	Positive
ERG	TIE1	0.52801	2.59E-32	Positive
FLI1	ICAM1	0.5065	1.83E-29	Positive
FLI1	A2M	0.63456	6.01E-50	Positive
FLI1	DLL4	0.53812	1.01E-33	Positive
FLI1	MARCO	0.48172	2.01E-26	Positive
FLI1	HGF	0.53757	1.21E-33	Positive
FLI1	ARRB1	0.5232	1.17E-31	Positive
FLI1	CYR61	0.4488	9.48E-23	Positive
FLI1	EDNRB	0.59983	1.82E-43	Positive
FLI1	RETN	0.42341	3.55E-20	Positive
FLI1	ACVRL1	0.70104	5.57E-65	Positive
FLI1	ENG	0.65288	1.04E-53	Positive
FLI1	FLT4	0.64726	1.57E-52	Positive
FLI1	NR4A3	0.46872	6.33E-25	Positive
FLI1	PTH1R	0.4162	1.74E-19	Positive
FLI1	TIE1	0.68268	1.99E-60	Positive
FLI1	ICAM2	0.68806	9.99E-62	Positive
FOS	NR4A1	0.57363	4.46E-39	Positive
FOXA2	DLL4	0.40778	1.07E-18	Positive
FOXA2	ARRB1	0.44232	4.51E-22	Positive
FOXA2	NR0B2	0.6318	2.12E-49	Positive
GATA6	ICAM1	0.46427	2.00E-24	Positive
GATA6	A2M	0.46541	1.49E-24	Positive
GATA6	ARRB1	0.40406	2.34E-18	Positive
GATA6	EDNRB	0.55237	8.54E-36	Positive
GATA6	BMP2	0.43468	2.71E-21	Positive
GATA6	ACVRL1	0.47913	4.04E-26	Positive
GATA6	FLT4	0.46192	3.63E-24	Positive
GATA6	NR4A3	0.48118	2.32E-26	Positive
GATA6	PTH1R	0.40226	3.41E-18	Positive
GATA6	TIE1	0.45951	6.69E-24	Positive
GATA6	ICAM2	0.44256	4.26E-22	Positive
NR4A1	CYR61	0.47941	3.74E-26	Positive
NR4A1	EDNRB	0.40127	4.19E-18	Positive
NR4A1	NR4A1	0.87655	2.51E-138	Positive
NR4A1	NR4A3	0.67207	6.04E-58	Positive
RXRG	NR0B2	0.51789	6.02E-31	Positive
RXRG	SSTR1	0.40021	5.22E-18	Positive
SNAI2	ARRB1	−0.4122	4.19E-19	Negative
SOX2	ICAM1	−0.453	3.35E-23	Negative
SOX2	WNT5A	0.43716	1.53E-21	Positive
SOX2	TNFRSF10C	−0.4192	8.97E-20	Negative
TCF21	SFTPD	0.46099	4.60E-24	Positive
TCF21	A2M	0.58905	1.30E-41	Positive
TCF21	DLL4	0.46154	4.00E-24	Positive
TCF21	MARCO	0.4991	1.57E-28	Positive
TCF21	HGF	0.4629	2.83E-24	Positive
TCF21	ARRB1	0.45689	1.29E-23	Positive
TCF21	EDNRB	0.60046	1.41E-43	Positive
TCF21	RETN	0.58495	6.35E-41	Positive
TCF21	ACVRL1	0.59241	3.50E-42	Positive
TCF21	AGTR2	0.51486	1.51E-30	Positive
TCF21	ENG	0.5005	1.05E-28	Positive
TCF21	FLT4	0.49341	7.93E-28	Positive
TCF21	PTH1R	0.54606	7.27E-35	Positive
TCF21	SCTR	0.47708	7.00E-26	Positive
TCF21	TIE1	0.57698	1.29E-39	Positive
TCF21	ICAM2	0.43744	1.43E-21	Positive
TP63	WNT5A	0.45232	4.00E-23	Positive
TP63	LTB4R	0.43924	9.36E-22	Positive
TP63	ARRB1	−0.4472	1.38E-22	Negative

### Establishment of the 10 DEIRGs-Based Prediction Model in LUSC

**Figure 5:**
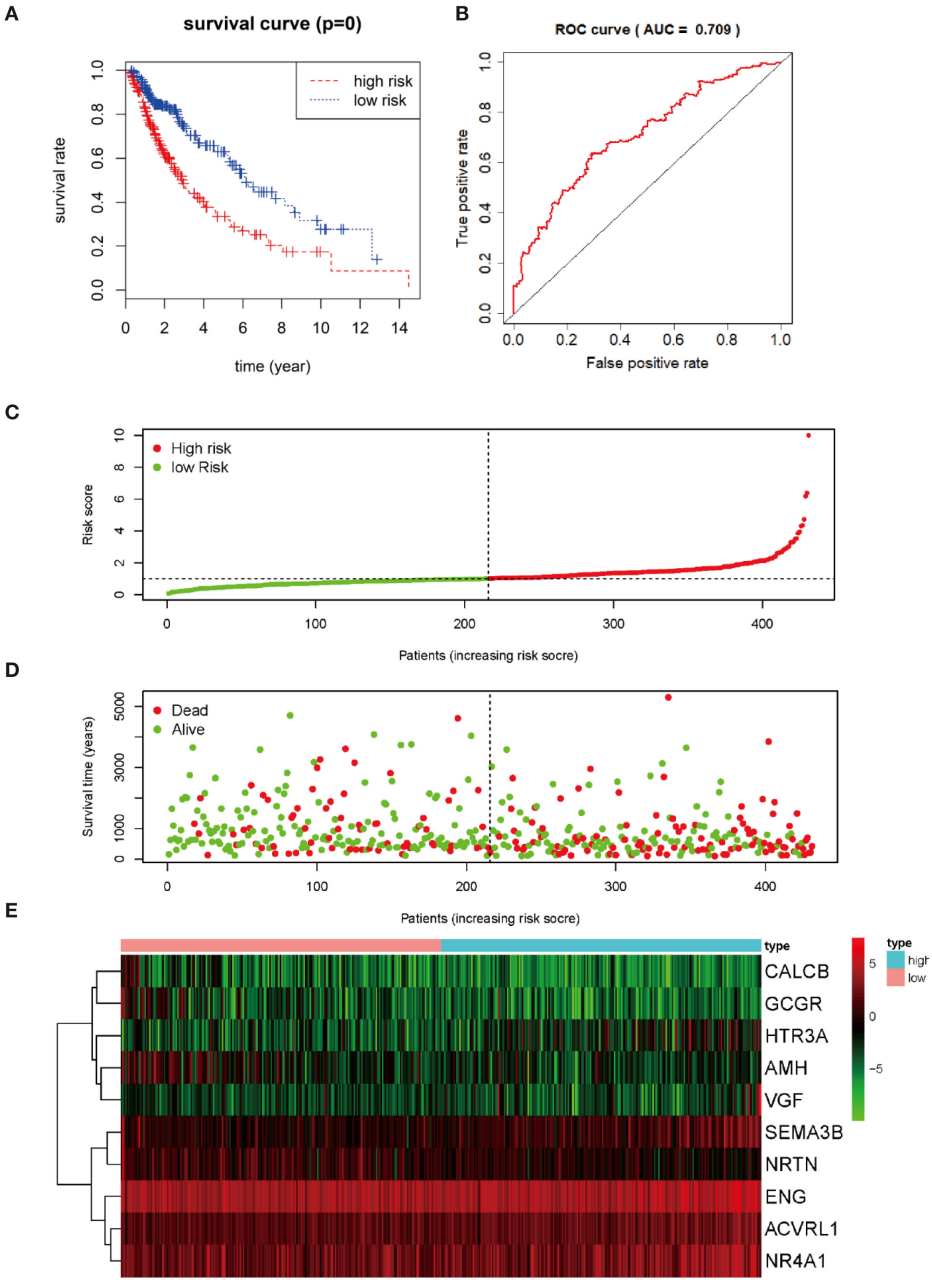
Prognosis value of 10 differential expression IRGs in LUSC patients. **(A)** Kaplan-Meier curve analysis for OS (overall survival) in LUSC patients using the 10 differential expression IRGs signature. **(B)** ROC curve analysis of the prognostic 10 differential expressions IRGs signature. **(C)** The risk score analysis of prognostic 10 differential expressions IRGs signature in LUSC high-risk group and low-risk group. **(D)** The survival status analysis of prognostic 10 differential expressions IRGs signature in LUSC high-risk group and low-risk group. **(E)** A risk heat-map constructed from 10 differential expression IRGs from 431 LUSC patients.

The prognostic DEIRGs were screened through univariate Cox regression analyses in LUSC, including 37 OS-related DEIRGs (*P* < 0.05) (Figure 4A). Then, the 37 DEIRGs were selected to incorporate into multivariate Cox regression analysis, which suggested that 10 DEIRGs might serve to be the prognostic factors to independently predict LUSC prognosis. These 10 DEIRGs were finally screened for constructing the prediction model (Table 6). Besides, expression profiles of these 10 DEIRGs were then linearly combined to build up the prediction model in LUSC. The weighted relative coefficients in multiple Cox regression were shown below: survival riskscore value = (0.2141 × SEMA3B expression + (−0.2698) × AMH expression + (−0.6777) × CALCB expression + 0.1896 × NRTN expression + 0.3628 × VGF expression + 0.4248 × ACVRL1 expression + (−0.3538) × ENG expression + (−0.4009) × GCGR expression + 0.1985 × HTR3A expression + 0.1339 × NR4A1 expression). Multivariate Cox regression analyses are shown in Table 6. Based on the median riskscore value, 431 cases who had intact survival time and status data were classified as high-(*n* = 215) or low- (*n* = 216) risk group. According to survival analysis based on the prediction model with the 10 DEIRGs, LUSC cases of high-risk group were associated with notably poor prognosis compared with those of low-risk group (*P* = 0) (Figure 5A). To evaluate the specificity and sensitivity of the prognostic model, we performed the ROC curve, for which the AUC value was 0.709, illustrating that the DEIRGs-based prediction model achieved better accuracy in survival monitoring (Figure 5B). The riskscore curve and survival status data of both groups of patients are exhibited in Figures 5C,D, respectively. As shown in Figure 5E, the expression of 10 DEIRGs was profiled.

**Table 6:** Multivariate Cox regression analyses of 10 DEIRGs in risk models in LUSC.

**IRG**	**coef**	**exp (coef)**	**se (coef)**	***z***	***P***
SEMA3B	0.2141	1.2387	0.093	2.3	0.021
AMH	−0.2698	0.7636	0.1251	−2.16	0.031
CALCB	−0.6777	0.5078	0.2912	−2.33	0.02
NRTN	−0.1896	0.8273	0.1092	−1.74	0.082
VGF	0.3628	1.4373	0.0903	4.02	5.90E-05
ACVRL1	0.4248	1.5293	0.2144	1.98	0.048
ENG	−0.3538	0.702	0.1871	−1.89	0.059
GCGR	−0.4009	0.6697	0.2073	−1.93	0.053
HTR3A	0.1985	1.2195	0.0856	2.32	0.02
NR4A1	0.1339	1.1433	0.0727	1.84	0.066

### Independent Prognosis Analysis

**Figure 6:**
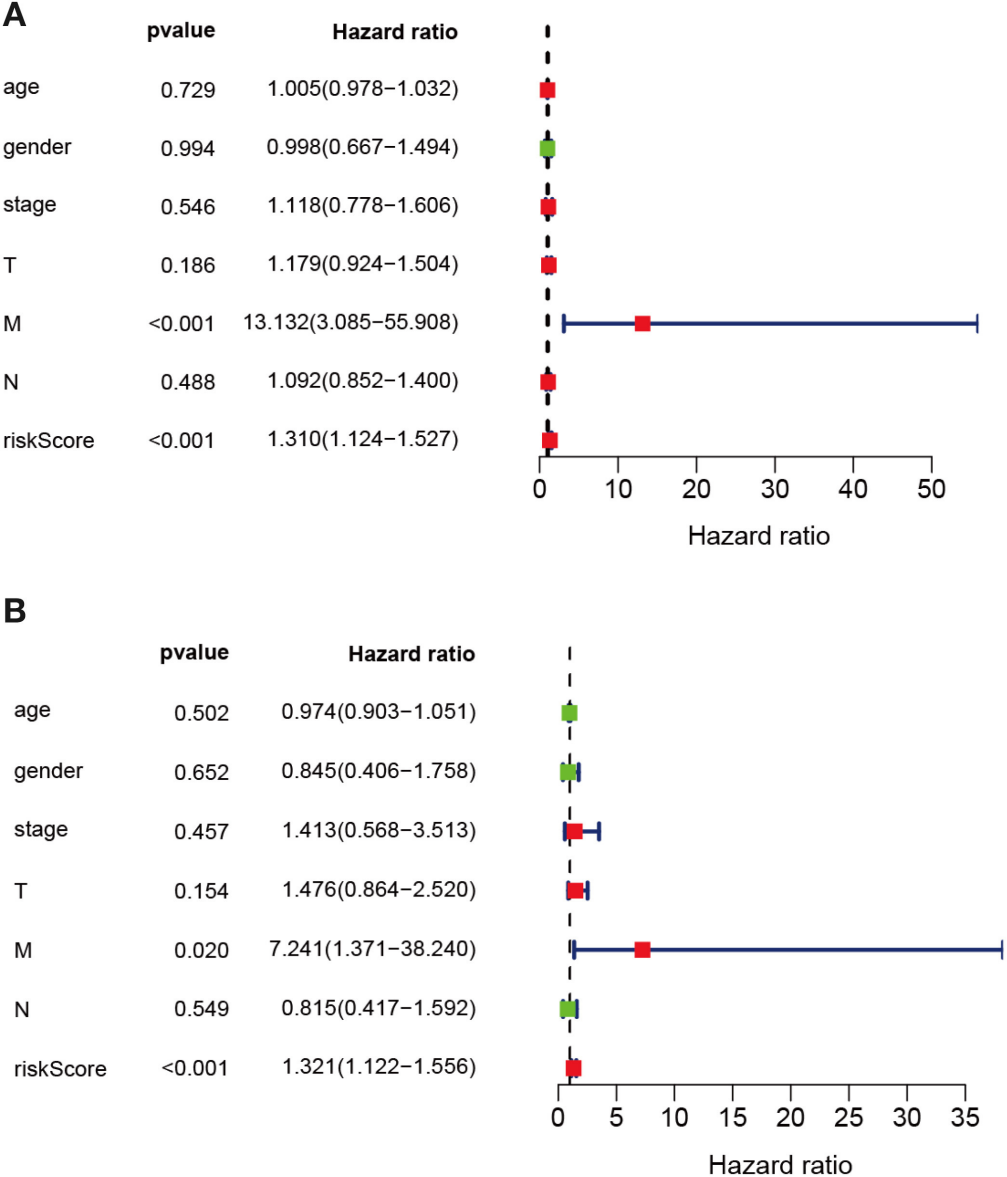
Univariate and multivariate independent prognostic analysis in LUSC. **(A)** Univariate independent prognostic analysis forest map of the prognostic immune-related genes model and LUSC clinicopathological characteristics. **(B)** Multivariate independent prognostic analysis forest map of prognostic immune-related genes model and LUSC clinicopathological characteristics. The red dots in the forest map shows that the clinical characteristic is a high-risk factor. The green dots in the forest map shows that the clinical characteristic is a low-risk factor.

The prognostic IRGs were screened for predicting the prognosis for LUSC cases. Eventually, altogether 37 DEIRGs showed significant correlation with overall survival (OS) (*P* < 0.05) (Figure 6A). Meanwhile, univariate independent prognostic analysis showed that, pathological M stage and the riskscore were related to OS, and the difference was of statistical significance (*P* < 0.001). Moreover, the multivariate independent prognostic analysis showed that, pathological M stage and the riskscore might serve as the independent prognostic factors to predict the survival for LUSC (Table 7; Figure 6B).

**Table 7:** Univariate and multivariate independent prognostic analysis of LUSC clinical characteristics based on prediction model.

**Variables**	**Univariate analysis**		**Multivariate analysis**	
	**Hazard ratio (95% CI)**	***p*-value**	**Hazard ratio (95% CI)**	***p*-value**
Age	1.005 (0.978–1.032)	0.729	0.974 (0.903–0.051)	0.502
Gender	0.998 (0.667–1.494)	0.994	0.845 (0.406–1.758)	0.652
Stage	1.118 (0.778–1.606)	0.546	1.413 (0.568–3.513)	0.457
T	1.179 (0.924–1.504)	0.186	1.476 (0.864–2.52)	0.154
M	13.132(3.085–55.908)	**<0.001**	7.241 (1.371–38.24)	**0.02**
N	1.092 (0.852–1.400)	0.488	0.815 (0.417–1.592)	0.549
riskScore	1.31 (1.124–1.527)	**<0.001**	1.321 (1.122–1.556)	**<0.001**

*The bold values represent that the prediction model of IRGs could be act as independent prognostic factors*.

### Relationships Between Differential IRGs in Prediction Models and Clinical Features in LUSC

**Figure 7:**
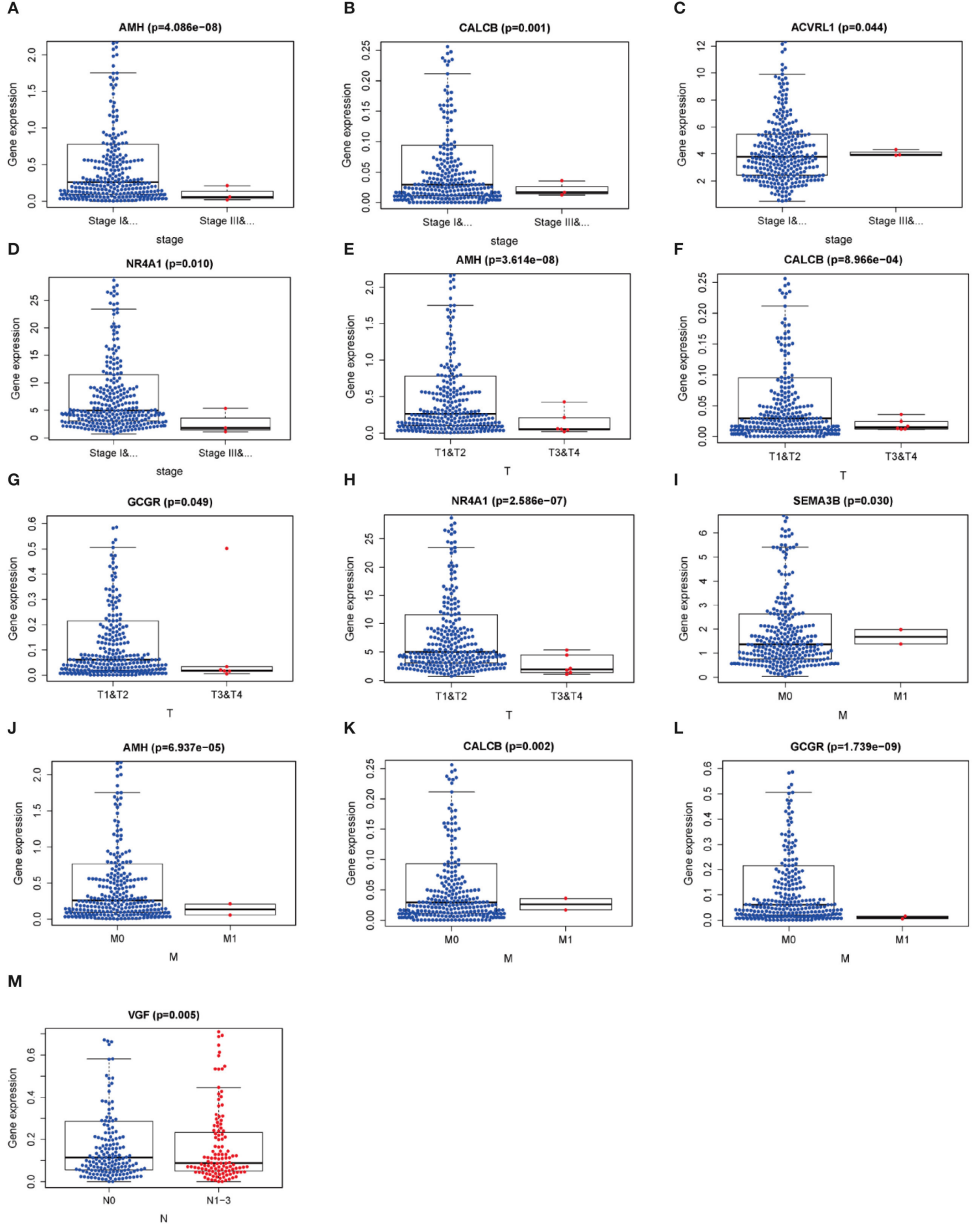
Relationships between the clinical-pathological characteristics and the expressions of differential expression IRGs in LUSC. **(A–D)** Differences in the expression of DEIRGs between the pathological TNM stages I-II/III-IV in LUSC. **(E–H)** Differences in the expression of DEIRGs between the pathological T1-T2/T3-T4 stages in LUSC. **(I–L)** Differences in the expression of DEIRGs between the pathological M0/M1 stages in LUSC. **(M)** Differences in the expression of DEIRGs between the pathological N0/N1-N3 stages in LUSC.

Relationships between DEIRGs in risk model and clinical characteristics, including gender, age, pathological classification, pathological T stage, pathological N stage, and pathological M stage, were analyzed (Table 8). As observed from Figure 7, the expression levels of AMH, CALCB, ACVRL1 and NR4A1 were significantly different in LUSC at pathological I-II stage compared with those at III-IV stage (*P* < 0.05) (Figures 7A–D). The expression levels of AMH, CALCB, GCGR, and NR4A1 were significantly different in LUSC at pathological T1-T2 stage compared with those at T3-T4 stage (*P* < 0.05) (Figures 7E–H). The expression levels of SEMA3B, AMH, CALCB, and GCGR were significantly different in LUSC at pathological M0 stage relative to those at M1 stage (*P* < 0.05) (Figures 7I–L). The expression of VGF was conspicuously different in LUSC at pathological N0 stage relative to that at N1-N3 stage (*P* = 0.005) (Figure 7M).

**Table 8:** Relationships between the expression of IRGs in risk models and the clinical characteristics in LUSC.

**Gene**	**Age (** **< =** **65/>65)**		**Gender (male/female)**		**Pathological Stage (I–II/III-IV)**		**T (T1-T2/T3-T4)**		**M (M0/M1)**		**N (N0/N1-N3)**	
	***t***	***P***	***t***	***P***	***t***	***P***	***t***	***P***	***t***	***P***	***T***	***P***
**SEMA3B**	−1.04	0.299	−1.319	0.188	0.15	0.055	0.92	0.381	2.776	**0.03**	−1.01	0.314
**AMH**	0.119	0.264	−0.12	0.905	6.389	**<0.001**	6.01	**<0.001**	5.744	**<0.001**	0.869	0.386
**CALCB**	−0.052	0.958	−0.426	0.671	3.29	**0.001**	3.355	**<0.001**	3.19	**0.002**	−0.65	0.519
NRTN	−1.363	0.175	−0.881	0.379	0.713	0.539	2.162	0.059	0.27	0.83	−1.26	0.212
**VGF**	0.413	0.68	0.37	0.712	0.23	0.834	1.406	0.184	−1.12	0.346	2.865	**0.005**
**ACVRL1**	1.063	0.289	0.977	0.33	2.164	**0.044**	0.662	0.533	1.582	0.19	0.011	0.991
ENG	1.035	0.301	0.763	0.447	0.272	0.809	−0.41	0.698	2.062	0.227	−0	0.998
**GCGR**	−0.042	0.967	0.828	0.409	0.79	0.501	2.264	**0.049**	6.217	**<0.001**	−0.7	0.482
HTR3A	−0.733	0.465	−0.179	0.858	−0.459	0.69	0.039	0.97	−0.64	0.637	−0.72	0.475
**NR4A1**	0.361	0.719	0.504	0.615	4.926	**0.01**	7.196	**<0.001**	3.44	0.12	−0.54	0.592
riskScore	−0.416	0.678	−0.266	0.79	−0.492	0.67	−0.236	0.821	−1.94	0.293	0.732	0.465

*The bold values represent the expression of IRGs were significantly associated with correspondence clinical characteristics*.

### Correlation Analysis Between DEIRGs in Prediction Model and Immunocyte Infiltration in LUSC

**Figure 8:**
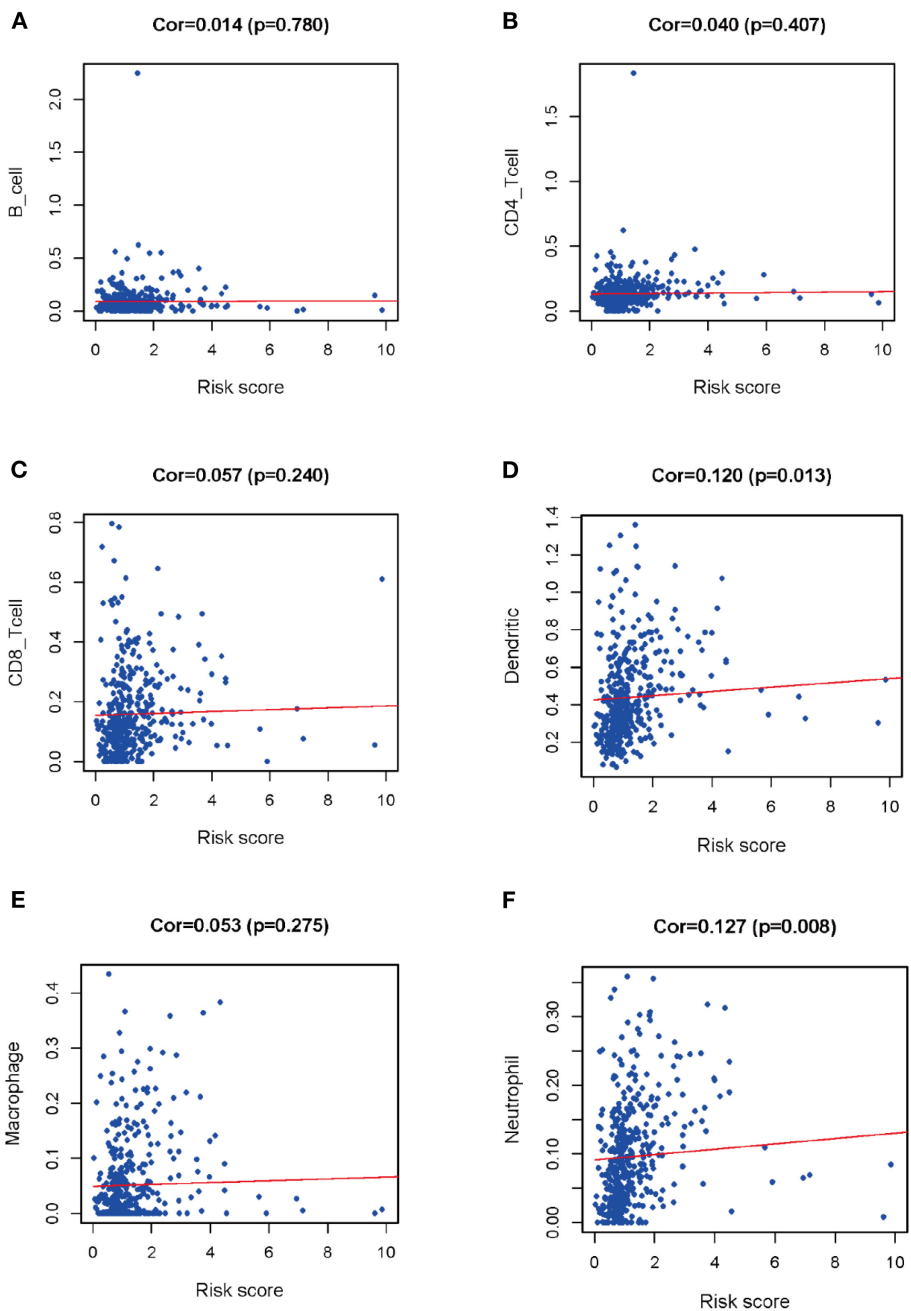
Relationships between prognostic value and degree of infiltration of six types of immune cells. **(A)** B cells; **(B)** CD4 T cells; **(C)** CD8 T cells; **(D)** Dendritic cells; **(E)** Macrophages; **(F)** Neutrophils.

To figure out whether DEIRGs precisely reflected the status of LUSC IME, correlation analysis was carried out to examine the relationship between DEIRGs in the LUSC prediction model and immunocyte infiltration (Figure 8). As shown in Figure 8, B cells, CD4-T cells, CD8-Tcells, Macrophages were not associated with the riskScore of the prediction model (*P* > 0.05) (Figures 8A–E). Dendritic cells and neutrophils had a positively relationship with DEIRGs in prediction model (*P* < 0.05) (Figures 8D,F).

## Discussion

In recent years, IRGs show increasing importance to cancer development and immunotherapies (24–27). However, transcriptome studies on IRGs, the relationships of IRGs with clinical characteristics, and the molecular mechanisms have not been performed yet. In the present work, the Cox prediction model was established for revealing IRGs specific to LUSC, so as to predict LUSC prognosis. The regulatory network between IRGs and TFs revealed the potential novel molecular mechanisms in LUSC. In this study, the DEIRGs obtained in LUSC might play a vital role in predicting the prognosis for LUSC. More importantly, an individualized Cox prediction model with DEIRGs was adopted for measuring immunocyte infiltration and evaluating the clinical prognosis.

In recent years, the prognostic or predictive biomarkers associated with the tumor IME are promising to identify new molecular targets and to enhance the treatment for patients during immunotherapy development (28–34). Several recent studies reveal the prognostic biomarkers in tumor IME for predicting tumor prognosis. Li et al. found that four IRGs were identified as the biomarkers to predict the prognosis for breast cancer (35). Pan et al. discovered that 149 immune genes were identified as the prognostic genes in tumor IME to predict ESCC prognosis (36). Yang et al. suggested that the diagnostic immune score (DIS) and prognostic immune score (PIS) showed diagnostic and prognostic significance for cancers in the digestive system (37). Nowadays, prognostic biomarkers related to the tumor IME in lung cancer are still lacking.

A study demonstrates that the NSCLC IME may be adopted to be the potential prognostic biomarkers to predict patient prognosis after receiving immune checkpoint inhibitor treatments (37). However, the molecular mechanism of prognosis-related DEIRGs associated with DETFs in LUSC tumor IME is not examined yet. The present study was carried out to explore DEIRGs and establish the IRGs-based prognostic model in LUSC IME to reveal the prognostic biomarkers to predict LUSC diagnosis and prognosis.

According to functional enrichment analysis in this work, DEIRGs showed the highest enrichment levels within tumor-related typical pathways, including the JAK-STAT signal transduction pathway, the TGF-β signal transduction pathway, the PI3K-Akt signal transduction pathway, the MAPK signal transduction pathway, and the TNF signal transduction pathway. According to recent research, alterations in MET-activation and JAK2-inactivation are the independent factors that affect the response to immune checkpoint inhibitors like PD-L1 in lung cancer (25). As suggested in one study, the combination of MEK and PD-L1 inhibitors in pre-clinical and *ex-vivo* NSCLC models exerts an important part in predicting patient sensitivity to such therapies (38).

For further exploring the underlying mechanisms of DEIRGs at molecular level, the TF-mediated IRGs network was constructed in the present study, so as to reveal the significant TFs regulating DEIRGs in this network. FOXA2, TP63, FLI1, TCF21, EPAS1, ERG, GATA6, FOS, CENPA, SOX2, RXRG, NR4A1, and SNAI2 were the DETFs that might regulate the DEIRGs in LUSC. Tang et al. discovered that curcumin inhibited the growth of human NCI-H292 LUSCs by up-regulating FOXA2 expression (39). FLI1 acts as a novel oncogenic diver to promote the metastasis of small cell lung cancer (SCLC). LncRNA LINC00163 serves as the tumor suppressor through transcriptionally up-regulating TCF21 expression in inhibiting the development of lung cancer (40). In addition, the hypoxic-stabilized EPAS1 proteins transactivate DNMT1, which further promotes the hypermethylation of EPAS1 promoter and down-regulates EPAS1 mRNA expression in NSCLC (41). A recent study showed that CENPA could act as a novel diagnostic biomarker in lung adenocarcinoma (42). Compared with previous studies, our study had first constructed the co-expression DETFs-prognosis-related DEIRGs regulatory network in LUSC using bioinformatics analysis. From the network in our study, the DETFs positively and negatively regulated the DEIRGs, which shed new light on exploring the DEIRGs mechanisms in LUSC at molecular level.

In our study, univariate as well as multivariate Cox regression analysis was carried out to constructed the DEIRGs-based Cox prediction model. Eventually, the 10 DEIRGs in prediction model played critical parts in predicting LUSC prognosis. In addition, the AUC was 0.709, while the *P*-value between high and low risk groups was 0, which indicated that the Cox prediction model might be able to accurately estimate LUSC prognosis. Univariate as well as multivariate independent prognosis analysis in our study indicated that, the pathological M stage and the riskScore of the Cox prediction model might serve as the independent prognostic factors to predict LUSC prognosis. Furthermore, 10 DEIRGs were applied for correlation analysis between the expression profiles of IRGs and clinical characteristics. Semaphorin 3B (SEMA3B) can be used to be the tumor suppressor gene to suppress the Akt signal transduction pathway, which is achieved via the neuropilin-1 receptor in lung cancer cells (43). The AMH/AMHR2 axis provides a novel insight to illustrate the TGF-β/BMP resistance-associated signaling in NSCLC (44). Epigenetic modifications facilitate the expression of VGF, up-regulate its protein expression, and promote epithelial-mesenchymal transition (EMT) progression as well as kinase inhibitor resistance within NSCLC (45). GCGR acts as a member of the prognostic model, which can exert an important part in predicting LUSC survival (46). In addition, NR4A1 exerts an important part in the regulation of TGFβ-induced invasion and migration of lung cancer cells (47).

Our study first used bioinformatic analysis to integrate the clinical characteristics of LUSC with the expression profiles of 10 DEIRGs to explore the statistically significant DEIRGs for forecasting LUSC diagnosis and prognosis. Finally, immunocyte correlation analysis was conducted using the contents of the TIMER database of six types of immunocytes. According to our results, dendritic cells and neutrophils exhibited a significantly positive regulatory relationship with the riskscore of the Cox prediction model. Compared with previous studies, the present study presented the new signature in which DEIRGs were selected as the center, which might be used to predict LUSC prognosis. Furthermore, the DEIRGs might act as the prognostic biomarkers and immune status monitor for predicting LUSC prognosis. We explored the relationships between DEIRGs in prediction model and immunocyte infiltration to reflect the status of IME in LUSC. Dendritic cells and neutrophils were positively correlated with DEIRGs in prediction model, which indicated that the high infiltration levels of dendritic cells as well as neutrophils might be identified in high-risk LUSC patients. These results showed that DEIRGs in prediction model could act as predictor for predicting immunocyte infiltration in LUSC. A study demonstrated that STAT3 and NF-κB signaling pathways were simultaneously attenuated in dendritic cells of lung cancer (48). A study showed that the tumor-associated CD66b neutrophils were correlated with adverse prognostic factors of NSCLC (49). The number of mature dendritic cells were positively correlated with survival time in NSCLC patients (50), While our findings showed that dendritic cells had a positively relationship with the riskScore of the prediction model. These results demonstrated that the prediction model based on DEIRGs could predict the status of immunocyte infiltration in LUSC.

Our prognostic model, which was constructed based on 10 DEIRGs in LUSC, indicated favorable clinical viability. It showed that DEIRGs performed moderately in the ROC curve, and were associated with age, gender, pathological TNM stage, and metastasis. This predictive model may provide a treatment plan based on the immunocyte infiltration degree revealed by DEIRGs.

In conclusion, our study identifies the DEGs, DEIRGs, and DETFs by bioinformatics analysis from TCGA, ImmPort and Cistrome databases. A TFs-IRGs network is performed to reveal the possible mechanism for DEIRGs in LUSC at molecular level. Additionally, the Cox prediction model is constructed for identifying the prognostic independent factors to predict LUSC prognosis. Immunocyte correlation analysis is also performed to identify the relationships between the immune status and the clinical outcomes for LUSC patients.

## Data Availability Statement

The datasets presented in this study can be found in online repositories. The names of the repository/repositories and accession number(s) can be found in the article/supplementary material.

## Author Contributions

Y-QQ and RL designed the research. J-PL, Y-HY, and XL analyzed the data. X-JZ and XC collected the literature. RL drafted the manuscript. Y-QQ revised the manuscript. All authors read and approved the final manuscript.

## Conflict of Interest

The authors declare that the research was conducted in the absence of any commercial or financial relationships that could be construed as a potential conflict of interest.

## Abbreviations

AUC: Area under the curves
BC: breast cancer
CRC: colorectal cancer
DAVID, The Database for Annotation: Visualization and Integrated Discovery
DEGs: Differentially expressed genes
DEIRGs: Differentially expressed immune-related genes
DETFs: Differentially expressed transcription factors
EMT: epithelial-mesenchymal transition
FDR: False discovery rate
GO: Gene ontology
IMMPORT: Immunology Database and Analysis Portal
IME: immune microenvironment
IRGs: Immune-related genes
KEGG: Kyoto Encyclopedia of Genes and Genomes
LUSC: Lung squamous cell carcinoma
NSCLC: Non-small-cell lung cancer
OS: overall survival
PCa: prostate cancer
ROC: Receiver operating characteristic
TCGA: The Cancer Genome Atlas Project
TIMER: Tumor Immune Estimation Resource
TFs: Transcription factors.
